# Impact of COVID-19 Pandemic on Health Behaviours of Adolescents Living in Italy: Data from 2021/2022 HBSC Survey

**DOI:** 10.3390/healthcare13162035

**Published:** 2025-08-18

**Authors:** Silvia Ciardullo, Daniela Pierannunzio, Paola Dalmasso, Giacomo Lazzeri, Alessio Vieno, Paola Nardone

**Affiliations:** 1 National Centre for Disease Prevention and Health Promotion, Istituto Superiore di Sanità (National Institute of Health), 00161 Rome, Italy; silvia.ciardullo@iss.it (S.C.); daniela.pierannunzio@iss.it (D.P.); 2 Department of Public Health and Pediatrics, University of Torino, 10126 Torino, Italy; paola.dalmasso@unito.it; 3 Department of Molecular and Developmental Medicine, University of Siena, 53100 Siena, Italy; giacomo.lazzeri@unisi.it; 4 Department of Developmental Psychology and Socialization, University of Padova, 35131 Padova, Italy; alessio.vieno@unipd.it

**Keywords:** adolescents’ health, COVID-19 pandemic impact, HBSC, well-being, social relationships

## Abstract

**Background**: Italy was among the earliest countries globally to be heavily impacted by the outbreak of the new coronavirus (SARS-CoV-2). During this period, schools were closed to students, and distance learning was adopted. The school closure has changed children’s and adolescent’s everyday lives, affecting their emotional resilience and mental health overall. **Objectives**: The aim of this study was to provide evidence regarding the influence of the pandemic period and lockdown measures on the well-being of adolescents living in Italy. The effects of COVID-19 were evaluated. **Methods**: The Health Behaviour in School Aged-Children (HBSC) 2021/2022 Italian data were used to describe changes in different dimensions of adolescents’ lives (e.g., family and peer relationships, mental health, school performance, physical activity, eating behaviour, and life in general). **Results**: Data from a sample of 89,321 adolescents participating in the 2021/22 HBSC wave were analysed. The areas where adolescents reported the greatest positive perceived change due to the pandemic period were family relationships (54.0%) and relationships with friends (44.7%). In contrast, negative changes were noted on mental health (41.1%), physical activity practice (42.9%), and life expectancy in general (37.2%). The effects of the coronavirus measures on family finances (48.9%), eating behaviour (43.6%), and overall health (43.7%) were most frequently assessed as neutral, i.e., neither positive nor negative. **Conclusions**: These results, in particular the adverse COVID-19 effects, reinforce the idea that during a pandemic, in addition to containing the infectious agent, specific attention must be paid to adolescents’ well-being, preserving their mental health and overall health.

## 1. Introduction

Italy was among the earliest countries globally to be heavily impacted by the outbreak of the new coronavirus (SARS-CoV-2), and it was the first country in Europe to implement a nationwide lockdown. Kindergartens, schools, and universities began to close at the end of February 2020, starting in northern Italy, and subsequently, on 10 March 2020, the government extended the restriction measures to all regions and autonomous provinces (AP) of the country. Children, teenagers, and their families experienced almost total isolation until 3 May 2020, and all schools remained closed until September of the same year [1]. According to the United Nations Educational, Scientific and Cultural Organization [2], in Italy, from 2020 to 2022, all schools were closed entirely for 93 days due to the isolation measures implemented, and 263 days if partial closures are also considered. During this period, the schools were closed to students and staff to contain the spread of the SARS-CoV-2 virus, and educational services were provided through remote learning [2].

Restrictive measures have changed children’s and adolescents’ everyday lives, affecting their emotional resilience and mental health overall [3,4,5,6]. Some authors reported that school closures reduced peer interaction and limited opportunities for physical activity [7,8], while social distancing led to isolation and loneliness, increasing mental health disorders [9]. Globally, teenagers have shown higher levels of depression, anxiety, and overall mental health decline due to the pandemic [10].

However, the experience of the pandemic was not homogeneous among all youth and across the countries [11]. Many studies report negative effects on children’s health behaviours, including decreased physical activity, increased sedentary time, media use, consumption of snacks and sweets, and sleep disorders [10,12,13,14,15]. In contrast, some studies reported an improvement of family relationships and support, and a decrease in conflict among adolescents [16,17]. The reduction in social and school responsibilities may have eased pressure on youth and parents, making the pandemic a source of relief for some families [10,18].

Adolescents across 22 countries and regions of the WHO European Region who took part in the 2021/2022 Health Behaviour in School-Aged Children (HBSC) survey reported that the COVID-19 pandemic had significant impacts across multiple areas of their lives; negative impacts most commonly were related to mental health, physical activity, and school performance, and positive impacts to relationships with family and friends [11].

In general, the COVID-19 pandemic and the associated lockdown and quarantine at home may have triggered adverse reactions among adolescents that could persist after the pandemic and may have a greater impact on more socioeconomically disadvantaged groups [19]. Consequently, the sociodemographic inequality gap might have been increased during this period [20].

Moreover, evidence showed differences in the COVID-19 pandemic’s impact by gender, age groups, and social class. The pandemic seemed to have deepened pre-existing disparities among social groups [21].

In this framework, the present study provides further evidence regarding the influence of the pandemic period and lockdown measures on the health of adolescents living in Italy. The HBSC 2021/2022 Italian database provides an opportunity to map and describe the various dimensions of adolescents’ and their families’ lives during the pandemic and in the subsequent months.

## 2. Materials and Methods

### 2.1. Study Population

The present study was based on data from the Italian Health Behaviour in School Aged-Children (HBSC) carried out during the 2021/2022 school year. The HBSC is an international, multicentre survey conducted every four years using a standardised research protocol to investigate health-related behaviours in adolescents [22]. Italy became a member of the HBSC international network in 2000, and since then, six data collection waves have been conducted [23]. Starting from 2010, each survey has been supported and financed by the Ministry of Health and coordinated by the Italian National Institute of Health (ISS), in collaboration with the Universities of Torino, Padova, and Siena [24].

HBSC is the first Italian population-based survey on adolescent behaviours, representative at national and regional levels and involving more than 80,000 students (aged 11, 13, 15 and, since 2022, 17-year-olds have also been included) recruited from school classes throughout all 21 Italian regions and AP [24,25]. The school class was the primary sampling unit, drawn by a stratified systematic cluster sampling from the list of all public and private schools obtained from the Ministry of Education.

#### Consent to Participate

Parents of the students were provided with an informational note outlining the purpose of the survey prior to data collection. Families could refuse participation by filling in the note and returning it to the involved classes’ teachers.

Data were collected in classroom settings through standardised, self-filled online questionnaires. Anonymity and confidentiality of all participants were ensured.

A comprehensive explanation of the objectives, theoretical basis, and methodology of the international and Italian HBSC study is available in another source [25,26].

### 2.2. Measures

#### 2.2.1. COVID-19 Impact Measures

To evaluate the impact of the pandemic on various aspects of students’ lives, the international HBSC coordination team developed the COVID-19 impact scale. The question was “Since the beginning of the Corona pandemic, many people’s lives have been affected (i.e., lockdowns, school closures, distance learning, contact restrictions, social distancing and hygiene rules, closures of leisure and sports facilities, travel restrictions). What impact have these measures had on the following aspects of your life?”. The scale comprises ten items on the impact of the COVID-19 measures on (1) life in general, (2) overall health, (3) relationships with family, (4) relationships with friends, (5) mental health, (6) school performance, (7) physical activity, (8) eating behaviour, (9) future expectations, and (10) family finances.

The answers were measured on a five-point Likert scale from “very negative” to “very positive,” with a neutral middle category [27,28]. The original categories have been reclassified: “very negative” and “fairly negative” as “negative impact,” “very positive” and “fairly positive” as “positive impact,” with the neutral category unchanged.

#### 2.2.2. Contextual Variables

The Family Affluence Scale (FAS) was used to evaluate perceived family wealth [28,29]; this scale consists of six items: holiday travel frequency, own bedroom, number of computers, family vehicle ownership, number of bathrooms, and presence of a dishwasher. Responses (0 = not at all, 1 = once, 2 = twice, and 3 = more than twice) were scored, summed, and categorised into three levels: low (≤6), medium (7–9), and high (≥10). The study collected the sociodemographic characteristics of parents, which included the area of residence (North, Central, and South), parental nationality (Italian or foreign; categorised as both Italian, one foreign parent, or two foreign parents), parents’ educational level (primary school, secondary school, high school, degree, master/doctorate/specialisation, or do not know; in the analyses the highest level between the two parents was used to classify them as follows: low = both parents with less than high school/ secondary school, medium = at least one of the parents with high school/secondary school school, and high = at least one of the parents with a university degree or higher).

### 2.3. Statistical Analysis

Differences in prevalence according to sociodemographic characteristics were tested by the Pearson-design-based X2.

Binary Logistic regression analyses were conducted to explore the relationship between COVID-19 impact variables and sociodemographic factors. The probability of negatively and positively perceiving the various aspects of the effect of COVID-19 was expressed as odds ratios (OR) with corresponding 95% confidence intervals (CI). All statistical analyses were performed using Stata version 18.0. Cases with missing data were omitted from the analysis.

## 3. Results

Data from a sample of 89,321 adolescents participating in the 2021/22 HBSC wave were analysed: 48.5% females, 25.0% aged 11, 26.3% aged 13, 25.6% aged 15, and 23.1% aged 17. Table 1 shows the sociodemographic characteristics of the sample. Most of the parents of adolescents had a medium level of education (37.1%), and 29.8% had at least a university degree, although 23.1% of the adolescents reported not knowing their parents’ level of education.

More than 8 out of 10 adolescents had both Italian parents, and only 9.8% had both foreign parents. The sample consisted of 51.5% of adolescents from a middle socioeconomic level.

- Assessment of the COVID-19 measures on various areas of life

**Figure 1:**
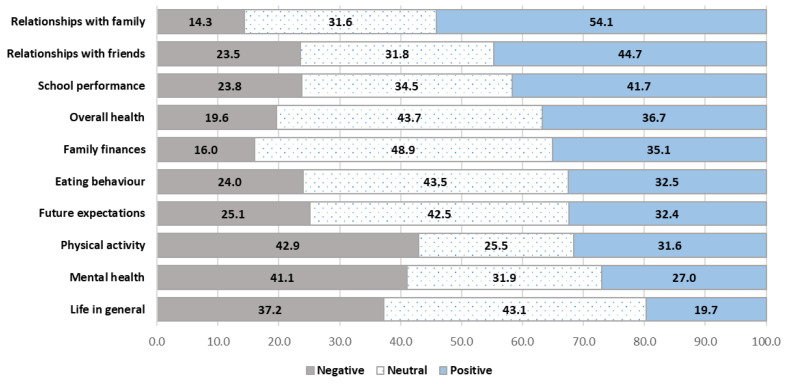
Perceived impact of the coronavirus measures on various areas of life.

The perceived impact of COVID-19 across the ten dimensions investigated (life in general, overall health, relationships with family, relationships with friends, mental health, school performance, physical activity levels, eating behaviour, future expectations, and family finances) was reported in Figure 1. The areas where adolescents showed the most significant positive change due to the pandemic period were family relationships (54.0%) and relationships with friends (44.7%). In contrast, negative changes were reported in mental health (41.1%) and physical activity practice (42.9%). The effects of the coronavirus measures on family finances (48.9%) and overall health (43.7%) were the most frequently assessed as neutral.

Table S1 (Supplementary Materials) shows the perceived impact of the pandemic on various areas of adolescents’ lives stratified by age, sex, geographical area of residence, parents’ educational level, and nationality, as well as FAS. Overall, younger students and boys were more likely to view the impact of COVID-19 measures positively compared with older students and girls. Significant gender differences were found in the perceived negative effects on mental health (girls: 52.4% vs. boys: 30.5%) and life in general (girls: 40.5% vs. boys: 34.2%). Additionally, negative perceptions of mental health increased with age, from 28.9% at age 11 to 53.2% at age 17.

Slight geographical differences emerged in the various areas of life investigated; in general, adolescents living in southern regions reported a more limited negative impact compared with their peers living in the North. Additionally, adolescents from low-income families experienced a significantly higher negative impact of COVID-19 measures.

Parents’ educational level was found to be a discriminating factor in students’ academic performance during the pandemic period. Youths with highly educated parents reported more positive changes in school performance during the pandemic (44.5%) than those with less educated parents (37.2%). Conversely, regarding improvements in eating habits, a higher percentage of youths with less educated parents (36.3%) experienced positive changes compared with those with more educated parents (29.3%).

In terms of parents’ nationality, students with both foreign parents were more likely to give a negative assessment of the COVID-19 measures than those with both Italian parents. In particular, marked differences were found in relationships with family, school performance, future expectations, family finances, as well as life in general.

- Logistic results

**Figure 2:**
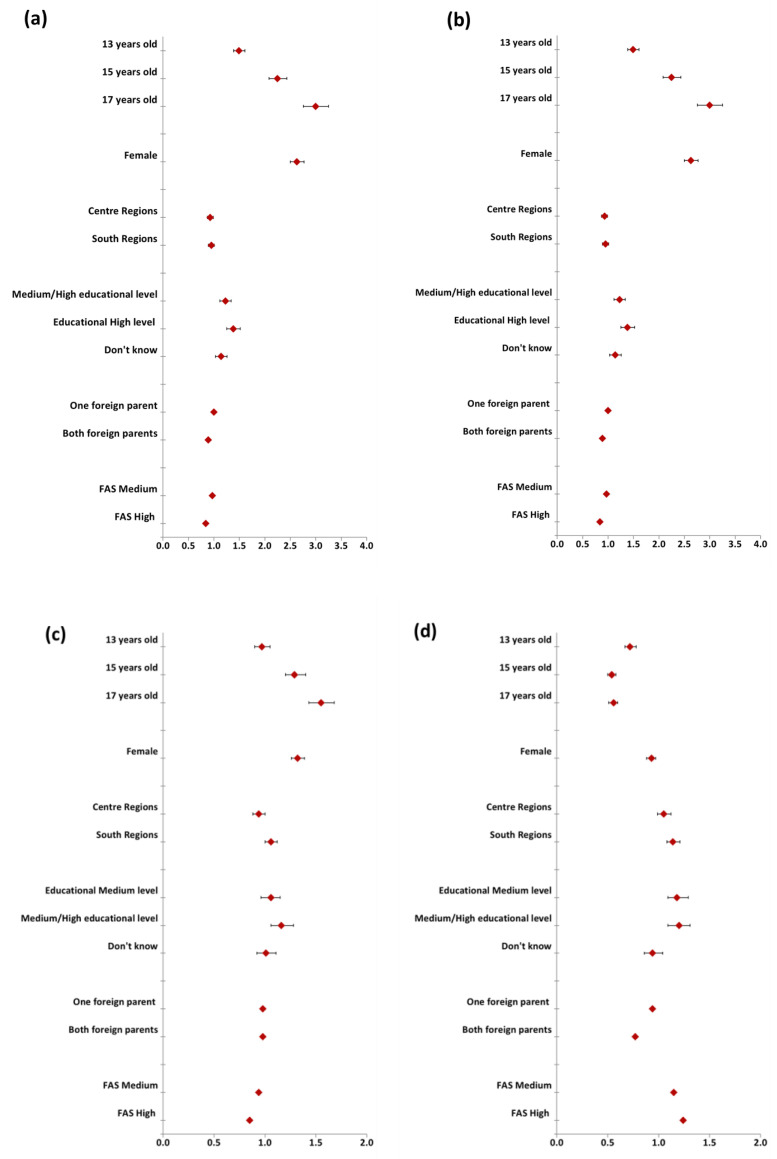
Perceived negative impact of the coronavirus measures on mental health (**a**) and life in general (**b**), logistic regression model. Perceived positive impact of the coronavirus measures on family relationship (**c**) and school performance (**d**), logistic regression model.

The risk of experiencing negative and positive effects of the coronavirus measures on various areas of life, according to age, sex, and context variables, was shown in Table S2 and Table S3, Supplementary Materials. Figure 2a–d, reported a visual summary of the regression model about sociodemographic factors and changes in four selected dimensions of adolescents’ lives.

In general, for most of the surveyed areas, being female and over 11 years old was a risk factor for the perception of their worsening due to the pandemic period.

As shown in Figure 2a,b, compared with 11-year-olds, 17-year-olds showed a significantly higher risk of a negative assessment of the effects of the coronavirus measures on mental health and life in general. In any case, among the 13–15 age group, the same significant trends observed in the 17-year-olds were also present, albeit to a slightly lesser extent.

Compared with boys, girls showed a significantly higher risk of a negative assessment of the effects of the coronavirus measures on mental health as well as life in general.

No significant differences were observed among adolescents living in different regions of Italy.

Respondents living with parents with a high educational level showed an increased risk of a negative assessment of the effects of the coronavirus measures on mental health and on life in general compared with those with parents with a low educational level. Respondents with high family affluence showed a significantly decreased risk of a negative assessment of the effects of the coronavirus measures in the area investigated compared with those with low family affluence.

The regression results for predicting the experience of positive influences of the coronavirus measures on relationship with family and school performance showed that the chances of a positive assessment were also unevenly distributed according to age, geographical distribution, and socioeconomic status (Figure 2c,d). From 13-year-olds to 17-year-olds, a significantly higher chance of a positive experience of the effects of the pandemic on relationships with family and school performance was observed. Girls showed a higher chance of experiencing the effects of the pandemic positively compared with boys in their relationships with family and school performance. Respondents living with parents with a high educational level showed an increased positive assessment of the effects of the coronavirus measures on the relationship with family compared with those with parents with a low educational level. These last results were not confirmed for the positive assessment of school performance due to the pandemic period.

## 4. Discussion

The objective of this study was to investigate the impact of the COVID-19 pandemic and lockdown measures on various aspects of adolescents’ lives, both in the short and long term.

Our study found that Italian adolescents participating in the 2021/2022 HBSC survey, two years after the pandemic began, reported both positive and negative effects on various aspects of their lives. A substantial number of adolescents experienced no impact. Specifically, 20–54% reported positive effects, 25–49% neutral, and 14–43% negative impacts related to the pandemic.

- *Comparison with international results*

The comparison of the Italian results with the international HBSC 2021/22 [11] pool analysis showed similar prevalence ranges of COVID-19 impacts on different areas of adolescents’ lives, even though the Italian HBSC results highlighted a lower number of Italian adolescents who claimed neutral impacts of the COVID-19 measures. Consequently, a greater prevalence of both positive and negative effects was observed among Italian adolescents compared to those from other countries [11].

In general, the Italian HBSC results concurred with those from the international HBSC in most areas of adolescent life explored. However, some differences were observed, suggesting possible differential effects of the pandemic measures among countries involved in the survey [11]. The Italian data revealed that of adolescents reporting positive impacts, most referred to relationships with their family and peers, as well as school performance. Although the improvement of family relationships is evidence highlighted in several other studies [11,17], another set of studies emphasises how the pandemic may have increased domestic conflicts [30]. The improvement in school performance appears to be a phenomenon confined to Italy, as it is not reflected in the international HBSC results [11].

Regarding the negative impacts, most Italian adolescents reported a deterioration in physical activity and mental health at a higher percentage compared to their international peers [11]. Adolescents from Italy were more likely to claim a negative impact of the coronavirus measures on their whole life with respect to those from other countries. In contrast to our results, a German HBSC 2021/2022 study reported an improvement in adolescents’ physical activity and a lower prevalence of young people who declared a positive impact of the pandemic on school improvement. In addition, a major negative effect of COVID-19 on mental health was observed among Italian adolescents than those from Germany [17].

Data stratified by the sociodemographic characteristics of adolescents and parents showed significant differences in adolescents’ life assessments. Younger adolescents, boys, those from southern Italy, and wealthier families mainly reported positive pandemic impacts on family and peer relationships and school performance.

Differences in individual areas of life according to age, gender, and perceived family wealth were also reported in the international report on the 2021/2022 HBSC survey and other studies [11,31]. In line with our results, at the international level, girls and adolescents from lower FAS families more often reported negative impacts in most areas of their lives, as well as boys and adolescents from higher FAS families more often claimed positive impacts [11]. It is known that individuals with a higher socioeconomic status have greater access to resources that promote healthy behaviours, whereas lower socioeconomic status is linked to unhealthy behaviours that may worsen during the pandemic [32]. The Italian results also showed a major improvement in school performance during the pandemic among adolescents with at least one parent with a medium or high educational level. This finding is in line with other studies that investigate the relationship between parents’ educational level and other health issues among children and adolescents [33,34]. Moreover, the pandemic measures had a more positive impact on the family relationships and school performance of adolescents with both Italian parents.

The spread of the SARS-CoV-2 virus was not uniform across the country; indeed, the northern regions were the most affected in terms of infections and hospitalisations [35]. Our data on the negative impact on adolescents’ physical activity and mental health showed no consistent geographical patterns; this result may indicate that the pandemic’s impact, especially on mental health, was largely independent of the spread of the virus.

While at the national level we did not find any geographical differences, the international literature reports evidence that some medium- and long-term effects of the pandemic on adolescents’ mental health and well-being have been more evident in low- and middle-income countries [36].

Overall, the COVID-19 health crisis has been linked to a notable decline in adolescents’ perceived well-being [37]. Indeed, the experience of the lockdown seems to have had a negative impact on their emotions and behaviours [30,38] and, more generally, their mental health [39].

The evidence produced by our study contributes to showing that the pandemic has had a greater negative impact on the most disadvantaged population subgroups, thereby widening health disparities [21,40].

- *Future research and implications*

To understand whether this disparity gap among young people can be compensated over the years, it is necessary to continue monitoring their health status through observational studies and targeted surveys on specific aspects of health, such as overall well-being and mental health [41]. A recent study [42] emphasises the importance of investing in adolescent health and well-being through education, healthcare, and nutrition, supported by laws and policies that empower youth in research and decision-making. Finally, as suggested at the international level [43], interventions should promote the five fundamental components that serve as protective factors during disasters: a sense of safety, calming, social connectedness, self-efficacy, and community efficacy, as well as hope. These components constitute an evidence-based approach designed to support well-being and resilience at both the individual and community levels.

### Strengths and Limitations

The HBSC study enables national and international comparisons and trend analysis. The protocol and measurement tools are reliable, and the data accurately represent adolescents within the relevant Italian age groups (11, 13, 15, and 17 years old). Moreover, the adolescents’ response rate, as well as school participation for the Italian HBSC survey, is very high: 97.3% and 88.8%, respectively.

The 2021/2022 survey also provides a special opportunity to examine the health situation of adolescents, considering the effects of the COVID-19 pandemic, and to compare results with other countries.

The HBSC methodology’s strengths and limitations have been described elsewhere [25,26]. Some limitations are inherent in the cross-sectional design, which does not enable determining the directionality of emerging associations. A significant limitation is that the data collection relies on self-reporting, which could lead to potential bias.

Another issue is the high number of “Do not know” responses among 11-year-olds regarding parents’ education, likely because this information is unfamiliar at that age.

The assessment of the COVID-19 impact statement is based on self-reported measures, which have been collected two years after the lockdown restrictions. This temporal shift might have contributed to a possible recall bias in the responses.

## 5. Conclusions

This study provides an overview of the perceived negative and positive effects of the coronavirus pandemic among adolescents living in Italy. In line with other studies, these findings show significant negative impacts on mental health and life in general, mostly among girls and older adolescents. Positive impacts were noted in family relationships and school performances.

These results, in particular the negative effects, reinforce the idea that during a global pandemic, in addition to the implementation of measures to contain the infectious agent, particular attention should be paid to individuals’ well-being, preserving their mental health and overall wellness without neglecting age, gender, and socioeconomic differences.

## Figures and Tables

**Table 1:** Sociodemographic characteristics of the sample involved in the study (ages considered 11, 13, 15, and 17 years old).

Sociodemographic Variables		N	%	CI 95%
**Age**				
	11 years old	21,489	25.0	23.2–26.9
	13 years old	23,077	26.3	24.4–28.2
	15 years old	22,187	25.6	23.8–27.5
	17 years old	22,568	23.1	21.5–24.9
**Adolescent’s sex**				
	Male	45,232	51.5	50.6–52.4
	Female	44,089	48.5	47.6–49.4
**Residence area**				
	North	46,301	34.9	33–36.8
	Centre	18,417	21.8	20.1–23.6
	South	24,603	43.3	41.1–45.6
Parents’ education level **				
	High level of education	26,323	29.8	28.9–30.7
	Medium level of education	33,943	37.1	36.3–37.9
	Low level of education	6878	10.0	9.5–10.6
	Do not know	20,889	23.1	22.3–24.0
**Parents’ nationality**				
	Both Italian parents	70,113	82.3	81.7–82.9
	At least one foreign parent	8178	7.9	7.6–8.2
	Both foreign parents	10,215	9.8	9.31–10.3
**Family Affluence Scale (FAS)**				
	High	17,964	18.3	17.8–18.9
	Medium	45,682	51.5	50.8–52.1
	Low	21,946	30.2	29.4–31.0

** The highest educational level of the two parents.

## Data Availability

The data presented in this study are available in accordance with the 2022 Italian HBSC data access policy. Requests should be directed to the Principal Investigator, Dr. Paola Nardone (paola.nardone@iss.it).

## Supplementary Materials

The following supporting information can be downloaded at: https://www.mdpi.com/article/10.3390/healthcare13162035/s1, Table S1: Results of the Chi-square test on the perception of impact of the coronavirus pandemic on various areas of life, differentiated by sex, Family Affluence Scale (FAS), and sociodemographic characteristics; Table S2: Logistic to predict negative influence of COVID-19 pandemic; Table S3: Logistic to predict positive influence of COVID-19 pandemic.
