# Supplementary material for: Impact of COVID-19 Pandemic on Health Behaviours of Adolescents Living in Italy: Data from 2021/2022 HBSC Survey
Source: Healthcare (Basel). 2025 Aug 18;13(16):2035. doi: 10.3390/healthcare13162035 (PMC12385748; doi:10.3390/healthcare13162035)
Supplement: Supplementary file 1 [file healthcare-13-02035-s001.zip › healthcare-3789754-supplementary.pdf]

Table S1. Results of the chi-square test on the perception of impact of the coronavirus pandemic on various dimensions of adolescents' lives, differentiated by sex, family affluence scale (FAS) and Socio-demographic characteristics.

**Relationships with family**			**Negative**	**Neutral**	**Positive**	***p* Value**
		Overall	*14.3 (13.9- 14.7)*	*31.7 (31-32.3)*	*54.0 (53.3-54.8)*	
	**Age**	11 years old	12.4 (11.7-13.2)	20.3 (19.4-21.2)	67.3 (66.2-68.4)	0.000
		13years old	15.0 (14.2-15.8)	29.0 (27.9-30.1)	56.0 (54.8-57.3)	
		15 years old	15.6 (14.7-16.4)	37.4 (36.3-38.5)	47.0 (45.8-48.3)	
		17 years old	14.1 (13.3-14.9)	40.5 (39.3-41.7)	45.4 (44.0-46.8)	
	**Adolescent's sex**	Male	11.6 (11.1-12.1)	31.5 (30.6-32.4)	56.9 (56.1-57.8)	0.000
		Female	17.1 (16.5-17.8)	31.8 (31.0-32.6)	51.1 (50.1-52.0)	
	**Residence area**	North	15.1 (14.6-15.6)	34.1 (33.3-34.9)	50.8 (49.9-51.7)	0.000
		Centre	14.4 (13.6-15.3)	32.8 (31.5-34.1)	52.8 (51.3-54.2)	
		South	13.6 (12.9-14.3)	29.1 (28.0-30.2)	57.3 (56.0-58.6)	
	**Parents' educational level****	High level of education	14.7 (14.0-15.4)	33.1 (32.1-34.2)	52.2 (51.9-54.0)	0.000
		Medium level of education	13.6 (13.0-14.2)	33.5 (32.5-34.4)	52.9 (51.9-54.0)	
		Low level of education	13.2 (12.0-14.6)	31.1 (29.3-33.0)	55.7 (53.6-57.7)	
		Don't know	15.4 (14.5-16.2)	27.3 (26.2-28.3)	57.3 (56.2-58.6)	
	**Parents' nationality**	One foreign parent	16.0 (14.6-17.5)	32.3 (30.6-34.1)	51.7 (49.8-53.6)	0.000
		Both Italian parents	13.7 (13.3-14.2)	31.9 (31.2-32.6)	54.4 (53.6-55.2)	
		Both foreign parents	18.0 (16.8-19.2)	29.5 (28.1-31.0)	52.5 (50.9-54.1)	
	**Family Affluence Scale (FAS)**	High	13.5 (12.7-14.4)	30.3 (29.1-31.5)	56.2 (54.8-57.5)	0.000
		Medium	13.5 (13.0-14.0)	32.9 (32.1-33.8)	53.6 (52.7-54.5)	
		Low	15.8 (15.0-16.5)	30.7 (29.7-31.7)	53.5 (52.7-54.5)	
**Relationships with friends**			**Negative**	**Neutral**	**Positive**	***p* Value**
		Overall	*23.5 (23.0-24.0)*	*31.8 (31.2-32.4)*	*44.7 (44.1-45.4)*	
	**Age**	11 years old	23.5 (22.6-24.6)	24.1 (23.1-25.0)	52.4 (51.1-53.6)	0.000
		13years old	22.5 (21.5-23.5)	30.5 (29.5-31.5)	47.0 (45.9-48.2)	
		15 years old	25.8 (24.8-26.9)	34.4 (33.4-35.4)	39.8 (38.5-41.1)	
		17 years old	21.9 (20.9-22.9)	38.8 (37.7-40.0)	39.3 (38.0-40.5)	
	**Adolescent's sex**	Male	21.8 (21.2-22.5)	31.0 (30.2-31.8)	47.2 (46.3-48.0)	0.000
		Female	25.2 (24.5-25.9)	32.7 (31.9-33.4)	42.1 (41.3-43.0)	
	**Residence area**	North	26.0 (25.3-26.6)	33.7 (33.0-34.4)	40.3 (39.5-41.2)	0.000
		Centre	23.2 (22.2-24.3)	32.4 (31.2-33.7)	44.4 (43.1-45.7)	
		South	21.6 (20.7-22.5)	30.0 (29.0-31.0)	48.4 (47.2-49.6)	
	**Parents' educational level****	High level of education	25.5 (24.6-26.4)	32.4 (31.5-33.4)	42.1 (41.0-43.2)	0.000
		Medium level of education	22.5 (21.8-23.2)	32.9 (32.1-33.8)	44.6 (43.6-45.5)	
		Low level of education	21.1 (19.7-22.7)	32.5 (30.8-34.2)	46.4 (44.6-48.3)	
		Don't know	23.6 (22.6-24.5)	29.1 (28.1-30.1)	47.3 (46.2-48.5)	
	**Parents' nationality**	One foreign parent	23.8 (22.3-25.4)	30.6 (28.9-32.3)	45.6 (43.7-47.5)	0.234
		Both Italian parents	23.3 (22.8-23.9)	31.9 (31.3-32.6)	44.8 (44.0-45.5)	
		Both foreign parents	24.5 (23.2-25.9)	32.0 (30.6-33.5)	43.5 (41.9-45.0)	
	**Affluence Scale (FAS)**	High	22.6 (21.6-23.7)	30.5 (29.4-31.7)	46.9 (45.5-48.2)	0.000
		Medium	24.1 (23.4-24.7)	32.3 (31.6-33.0)	43.6 (42.8-44.5)	
		Low	22.9 (22.1-23.9)	32.1 (31.1-32.2)	45.0 (43.8-46.1)	

Physical activity			Negative	Neutral	Positive	*p* Value
		Overall	*42.9 (42.3-43.5)*	*25.5 (25.0-26.0)*	*31.6 (31.0-32.2)*	
	**Age**	11 years old	39.1 (38.0-40.3)	22.1 (21.2-23.0)	38.8 (37.7-39.9)	0.000
		13years old	42.9 (41.8-44.1)	25.9 (25.0-26.9)	31.2 (30.1-32.2)	
		15 years old	46.3 (45.0-47.6)	26.3 (25.4-27.3)	27.4 (26.3-28.4)	
		17 years old	43.2 (42.0-43.5)	27.8 (26.9-28.7)	29.0 (27.9-30.2)	
	**Adolescent's sex**	Male	42.0 (41.2-42.9)	23.5 (22.8-24.1)	34.5 (33.7-35.3)	0.000
		Female	43.9 (43.0-44.7)	27.7 (27.0-28.4)	28.5 (27.7-29.3)	
	**Residence area**	North	42.7 (41.9-43.5)	26.2 (25.6-26.8)	31.1 (30.4-31.8)	0.109
		Centre	42.4 (41.3-43.6)	25.9 (24.9-27.0)	31.7 (30.6-32.8)	
		South	43.3 (42.2-44.4)	24.7 (23.9-25.5)	32.0 (30.9-33.0)	
	**Parents' educational level****	High level of education	44.1 (43.1-45.2)	24.1 (23.2-24.9)	31.8 (30.8-32.8)	0.000
		Medium level of education	43.0 (42.1-43.9)	26.6 (25.9-27.4)	30.4 (29.6-31.2)	
		Low level of education	41.8 (39.9-43.8)	27.0 (25.4-28.6)	31.2 (29.4-33.1)	
		Don't know	41.8 (40.7-43.0)	24.8 (23.8-25.8)	33.4 (32.3-34.5)	
	**Parents' nationality**	One foreign parent	42.2 (40.4-44.0)	25.3 (23.7-27.0)	32.5 (30.8-34.3)	0.103
		Both Italian parents	43.1 (42.4-43.8)	25.3 (24.8-25.8)	31.6 (30.9-32.2)	
		Both foreign parents	42.3 (40.7-44.0)	27.2 (25.8-28.7)	30.5 (29.0-31.9)	
	**Family Affluence Scale (FAS)**	High	39.5 (38.3-40.8)	23.3 (22.4-24.4)	37.2 (35.9-38.4)	0.000
		Medium	43.8 (43.0-44.6)	24.9 (24.3-25.6)	31.3 (30.5-32.0)	
		Low	44.0 (42.9-45.1)	27.9 (27.0-28.8)	28.1 (27.1-29.1)	
**Mental Health**			**Negative**	**Neutral**	**Positive**	***p* Value**
		Overall	*41.1 (40.3-41.9)*	*31.9 (31.4-32.4)*	*27.0 (26.7-27.7)*	
	**Age**	11 years old	28.9 (27.9-29.9)	31.1 (30.2-32.1)	40.0 (38.9-41.1)	0.000
		13years old	37.0 (36.0-38.1)	33.3 (32.3-34.1)	29.7 (28.6-30.7)	
		15 years old	46.2 (44.7-47.5)	33.0 (32.0-34.1)	20.8 (19.8-21.9)	
		17 years old	53.2 (51.7-54.7)	29.9 (28.7-31.0)	16.9 (16.0-17.9)	
	**Adolescent's sex**	Male	30.5 (29.7-31.3)	35.7 (35.0-36.4)	33.8 (33.0-34.7)	0.000
		Female	52.4 (51.3-53.3)	27.8 (27.1-28.6)	19.8 (19.1-20.6)	
	**Residence area**	North	41.4 (40.4-42.5)	32.2 (31.5-32.9)	26.4 (25.5-27.3)	0.243
		Centre	41.0 (39.4-42.5)	32.5 (31.4-33.6)	26.5 (25.3-27.9)	
		South	40.9 (39.6-42.2)	31.3 (30.4-32.2)	27.8 (26.6-29.0)	
	**Parents' educational level****	High level of education	43.3 (42.2-44.5)	31.2 (30.2-32.1)	25.5 (24.5-26.6)	0.000
		Medium level of education	44.1 (43.1-45.2)	31.9 (31.0-32.7)	24.0 (23.1-24.9)	
		Low level of education	40.0 (38.1-41.9)	33.8 (32.0-35.5)	26.2 (24.5-28.0)	
		Don't know	33.9 (32.8-35.0)	32.2 (31.2-33.2)	33.9 (32.8-35.1)	
	**Parents' nationality**	One foreign parent	41.5 (40.7-42.3)	30.4 (28.7-32.2)	28.1 (26.4-30.0)	0.000
		Both Italian parents	41.5 (40.7-42.3)	32.2 (31.6-32.8)	26.3 (25.6-27.0)	
		Both foreign parents	37.8 (36.3-39.4)	30.8 (29.4-32.2)	31.4 (29.9-32.9)	
	**Family Affluence Scale (FAS)**	High	37.8 (36.5-39.2)	32.0 (30.8-33.2)	30.2 (29.0-31.4)	0.000
		Medium	42.0 (41.1-43.0)	31.8 (31.1-32.5)	26.2 (25.3-27.0)	
		Low	41.9 (40.8-43.0)	32.0 (31.1-33.0)	26.1 (25.1-27.1)	
**School performance**			**Negative**	**Neutral**	**Positive**	***p* Value**
		Overall	*23.8 (23.2-24.3)*	*34.5 (34.0-35.1)*	*41.7 (41.1-42.4)*	

	**Age**	11 years old	17.6 (16.7-18.5)	32.8 (31.8-33.9)	49.6 (48.4-50.8)	0.000
		13years old	22.1 (21.1-23.1)	34.9 (33.8-36.0)	43.0 (41.8-44.2)	
		15 years old	28.4 (27.2-29.5)	35.0 (33.9-36.1)	36.6 (35.4-37.9)	
		17 years old	27.2 (26.2-28.3)	35.3 (34.2-36.4)	37.5 (36.3-38.6)	
	**Adolescent's sex**	Male	24.1 (23.4-24.9)	33.3 (32.6-34.1)	42.6 (41.7-43.4)	0.000
		Female	23.4 (22.7-24.1)	35.8 (35.0-36.5)	40.8 (40.0-41.7)	
	**Residence area**	North	24.7 (24.0-25.5)	35.0 (34.3-35.7)	40.3 (39.4-41.2)	0.000
		Centre	22.8 (21.7-24.0)	36.1 (35.0-37.2)	41.1 (39.8-42.4)	
		South	23.5 (22.5-24.5)	33.3 (32.4-34.3)	43.2 (42.0-44.3)	
	**Parents' educational level****	High level of education	21.8 (20.9-22.7)	33.7 (32.8-34.7)	44.5 (43.3-45.6)	0.000
		Medium level of education	24.1 (23.3-25.0)	34.4 (33.5-35.2)	41.5 (40.5-42.4)	
		Low level of education	27.5 (25.9-29.3)	35.3 (33.6-37.1)	37.2 (35.5-38.9)	
		Don't know	24.1 (23.1-25.0)	35.4 (34.4-36.5)	40.5 (39.3-41.7)	
	**Parents' nationality**	One foreign parent	26.8 (25.2-28.5)	32.2 (30.5-33.9)	41.0 (39.2-42.8)	0.000
		Both Italian parents	22.7 (22.1-23.3)	34.6 (34.0-35.2)	42.7 (42.0-43.4)	
		Both foreign parents	30.2 (28.8-31.6)	35.6 (34.2-37.0)	34.2 (32.8-35.7)	
	**Family Affluence Scale (FAS)**	High	21.5 (20.5-22.5)	33.3 (32.1-34.4)	45.2 (44.0-46.6)	0.000
		Medium	22.8 (22.1-23.5)	34.7 (33.9-35.5)	42.5 (41.7-43.4)	
		Low	26.7 (25.8-27.8)	35.1 (34.1-36.2)	38.2 (37.1-39.3)	
**Future expectations**			**Negative**	**Neutral**	**Positive**	***p* Value**
		Overall	*25.1 (24.5-25.6)*	*42.5 (41.9-43.1)*	*32.4 (31.7-33.1)*	
	**Age**	11 years old	18.5 (17.6-19.4)	36.9 (35.8-38.1)	44.6 (43.4-45.7)	0.000
		13years old	22.3 (21.4-23.2)	42.0 (40.9-43.2)	35.7 (34.4-36.9)	
		15 years old	28.1 (27.0-29.2)	46.0 (44.9-47.2)	25.9 (24.7-27.0)	
		17 years old	31.9 (30.7-33.1)	45.2 (44.1-46.4)	22.9 (21.7-24.09	
	**Adolescent's sex**	Male	21.4 (20.8-22.1)	43.5 (42.6-44.3)	35.1 (34.2-36.0)	0.000
		Female	29.0 (28.1-29.8)	41.5 (40.8-42.4)	29.5 (28.6-30.3)	
	**Residence area**	North	27.2 (26.4-28.0)	44.4 (43.7-45.2)	28.4 (27.5-29.2)	0.000
		Centre	24.5 (23.5-25.6)	44.5 (43.3-45.7)	31.0 (29.8-32.3)	
		South	23.6 (22.7-24.6)	40.1 (39.0-41.1)	36.3 (35.1-37.6)	
	**Parents' educational level****	High level of education	24.7 (23.8-25.7)	44.1 (43.1-45.2)	31.2 (30.1-32.3)	0.000
		Medium level of education	25.7 (24.8-26.5)	43.2 (42.3-44.1)	31.1 (30.2-32.1)	
		Low level of education	25.3 (23.7-27.0)	42.0 (40.1-43.8)	32.7 (30.9-34.7)	
		Don't know	24.5 (23.5-25.5)	39.9 (38.8-41.0)	35.6 (34.5-36.7)	
	**Parents' nationality**	One foreign parent	27.0 (25.3-28.7)	41.3 (39.4-43.3)	31.7 (29.9-33.6)	0.000
		Both Italian parents	24.5 (23.9-25.2)	43.1 (42.4-43.7)	32.4 (31.6-33.2)	
		Both foreign parents	28.4 (27.0-29.8)	39.5 (38.0-41.0)	32.1 (30.7-33.6)	
	**Family Affluence Scale (FAS)**	High	22.4 (21.4-23.5)	41.9 (40.7-43.1)	35.7 (34.5-37.0)	0.000
		Medium	24.9 (24.2-25.7)	43.6 (42.8-44.4)	31.5 (30.6-32.3)	
		Low	26.9 (25.9-27.9)	41.6 (40.5-42.7)	31.5 (30.5-32.6)	
**Eating behaviour**			**Negative**	**Neutral**	**Positive**	***p* Value**
		Overall	*24.0 (23.5-24.6)*	*43.6 (42.9-44.1)*	*32.4 (31.8-33.1)*	
	**Age**	11 years old	18.9 (18.0-19.9)	38.6 (37.4-39.8)	42.5 (41.3-43.6)	0.000
		13years old	23.2 (22.2-24.1)	42.6 (41.6-43.7)	34.2 (33.1-35.3)	
		15 years old	27.0 (25.9-28.0)	45.1 (44.0-46.4)	27.9 (26.8-29.0)	

		17 years old	27.2 (26.2-28.3)	48.0 (46.8-49.2)	24.8 (23.7-26.0)	
	**Adolescent's sex**	Male	19.3 (18.7-20.0)	44.7 (43.8-45.5)	36.0 (35.2-36.9)	0.000
		Female	29.0 (28.2-29.8)	42.3 (41.6-43.1)	28.7 (27.9-29.5)	
	**Residence area**	North	23.9 (23.2-24.5)	46.3 (45.6-47.2)	29.8 (29.0-30.6)	0.000
		Centre	24.2 (23.1-25.3)	44.5 (43.3-45.8)	31.3 (30.0-32.6)	
		South	24.1 (23.2-25.1)	40.7 (39.7-41.7)	35.2 (34.1-36.3)	
	**Parents' educational level****	High level of education	25.0 (24.1-26.0)	45.7 (44.6-46.7)	29.3 (28.4-30.3)	0.000
		Medium level of education	24.9 (24.1-25.7)	44.8 (43.9-45.8)	30.3 (29.4-31.2)	
		Low level of education	23.9 (22.4-25.5)	39.8 (38.0-41.6)	36.3 (34.4-38.2)	
		Don't know	21.5 (20.6-22.5)	40.5 (39.3-41.6)	38.0 (36.9-39.2)	
	**Parents' nationality**	One foreign parent	23.1 (21.6-24.7)	44.6 (42.8-46.4)	32.3 (30.6-34.1)	0.000
		Both Italian parents	24.0 (23.4-24.6)	44.0 (43.3-44.7)	32.0 (31.3-32.7)	
		Both foreign parents	25.0 (23.7-26.4)	39.2 (37.7-40.8)	35.8 (34.3-37.3)	
	**Family Affluence Scale (FAS)**	High	22.8 (21.8-23.8)	43.3 (42.1-44.6)	33.9 (32.7-35.2)	0.000
		Medium	23.9 (23.3-24.6)	45.1 (44.2-45.8)	31.0 (30.3-31.8)	
		Low	24.9 (23.9-25.8)	41.6 (40.5-42.7)	33.5 (32.5-34.6)	
**Overall health**			**Negative**	**Neutral**	**Positive**	***p* Value**
		Overall	*19.6 (19.2-20.1)*	*43.7 (43.0-44.4)*	*36.7 (35.9-37.4)*	
	**Age**	11 years old	16.9 (16.0-17.8)	31.0 (30.0-32.1)	52.1 (50.9-53.3)	0.000
		13years old	17.0 (16.1-17.9)	42.6 (41.5-43.8)	40.4 (39.1-41.6)	
		15 years old	21.0 (20.1-22.0)	49.9 (48.7-51.0)	29.1 (28.0-30.3)	
		17 years old	24.1 (23.1-25.0)	51.7 (50.6-52.9)	24.2 (23.1-25.4)	
	**Adolescent's s**	Male	17.7 (17.1-18.2)	43.2 (42.4-44.2)	39.1 (38.1-40.0)	0.000
		Female	21.7 (21.0-22.5)	44.2 (43.3-45.0)	34.1 (33.2-35.1)	
	**Residence area**	North	20.0 (19.4-20.6)	45.2 (44.3-46.0)	34.8 (33.8-35.8)	0.000
		Centre	19.4 (18.5-20.4)	44.8 (43.4-46.2)	35.8 (34.4-37.2)	
		South	19.5 (18.7-20.3)	41.9 (40.7-43.2)	38.6 (37.2-40.0)	
	**Parents' educational level****	High level of education	18.9 (18.1-19.8)	46.9 (45.8-47.9)	34.2 (33.1-35.3)	0.000
		Medium level of education	19.7 (19.0-20.5)	46.9 (46.0-47.9)	33.4 (32.4-34.4)	
		Low level of education	18.7 (17.4-20.2)	43.3 (41.5-45.1)	38.0 (36.1-39.9)	
		Don't know	20.6 (19.8-21.5)	34.9 (33.8-36.1)	44.5 (43.3-45.6)	
	**Parents' nationality**	One foreign parent	19.3 (17.9-20.8)	43.5 (41.6-45.4)	37.2 (35.4-39.1)	0.000
		Both Italian parents	19.3 (18.8-19.9)	44.5 (43.7-45.2)	36.2 (35.4-37.0)	
		Both foreign parents	22.4 (21.1-23.7)	38.1 (36.5-39.7)	39.5 (38.0-41.1)	
	**Family Affluence Scale (FAS)**	High	17.9 (17.0-18.9)	42.6 (41.3-43.9)	39.5 (38.2-40.9)	0.000
		Medium	19.7 (19.1-20.4)	45.2 (44.3-46.0)	35.1 (34.2-36.0)	
		Low	20.6 (19.7-21.4)	42.4 (41.2-43.5)	37.1 (35.9-38.3)	
**Family finances**			**Negative**	**Neutral**	**Positive**	***p* Value**
		Overall	*16.0 (15.5-16.4)*	*48.9 (48.2-49.6)*	*35.1 (34.4-35.9)*	
	**Age**	11 years old	12.7 (12.0-13.5)	36.4 (35.3-37.5)	50.9 (49.7-52.0)	0.000
		13years old	14.0 (13.3-14.8)	47.0 (45.8-48.2)	39.0 (37.8-40.1)	
		15 years old	17.8 (17.0-18.6)	54.0 (52.7-55.3)	28.2 (27.1-29.4)	
		17 years old	19.5 (18.7-20.4)	58.6 (57.4-59.7)	21.9 (20.9-22.9)	

	**Adolescent's sex**	Male	15.7 (15.2-16.3)	47.8 (46.8-48.6)	36.5 (35.6-37.5)	0.000
		Female	16.2 (15.6-16.8)	50.1 (49.2-51.0)	33.7 (32.8-34.6)	
	**Residence area**	North	16.3 (15.7-16.9)	51.2 (50.3-52.1)	32.5 (31.5-33.5)	0.000
		Centre	16.7 (15.8-17.6)	49.8 (48.5-51.2)	33.5 (32.1-34.9)	
		South	15.3 (14.6-16.1)	46.6 (45.3-47.8)	38.1 (36.8-39.5)	
	**Parents' educational level****	High level of education	12.3 (11.6-13.0)	51.2 (50.0-52.4)	36.5 (35.4-37.7)	0.000
		Medium level of education	17.6 (16.9-18.2)	50.9 (50.0-51.9)	31.5 (30.5-32.4)	
		Low level of education	19.6 (18.2-21.1)	47.8 (45.9-49.7)	32.6 (30.8-34.5)	
		Don't know	16.4 (15.6-17.3)	43.5 (42.3-44.6)	40.1 (38.9-41.4)	
	**Parents' nationality**	One foreign parent	17.4 (16.0-18.9)	47.2 (45.3-49.2)	35.4 (33.5-37.3)	0.000
		Both Italian parents	15.1 (14.6-15.5)	49.8 (49.1-50.6)	35.1 (34.3-35.9)	
		Both foreign parents	22.0 (20.7-23.4)	43.0 (41.4-44.5)	35.0 (33.5-36.6)	
	**Family Affluence Scale (FAS)**	High	10.7 (9.9-11.4)	44.5 (43.1-45.8)	44.8 (43.5-46.2)	0.000
		Medium	14.4 (13.9-14.9)	51.3 (50.4-52.2)	34.3 (33.4-35.2)	
		Low	21.7 (20.9-22.7)	48.3 (47.2-49.4)	30.0 (28.9-31.1)	
**Life in general**			**Negative**	**Neutral**	**Positive**	***p* Value**
		Overall	*37.2 (36.6-37.9)*	*43.1 (42.5-43.7)*	*19.7 (19.2-20.2)*	
	**Age**	11 years old	33.4 (32.2-34.6)	40.7 (39.6-41.9)	25.9 (24.9-26.9)	0.000
		13 years old	32.8 (31.8-33.9)	45.8 (44.6-46.8)	21.4 (20.5-22.4)	
		15 years old	39.4 (38.2-40.6)	44.1 (42.9-45.3)	16.5 (15.7-17.4)	
		17 years old	44.0 (42.7-45.3)	41.5 (40.4-42.7)	14.5 (13.6-15.4)	
	**Adolescent's sex**	Male	34.2 (33.4-34.9)	42.8 (42.0-43.6)	23.0 (22.4-23.7)	0.000
		Female	40.5 (39.6-41.4)	43.4 (42.6-44.2)	16.1 (15.5-16.7)	
	**Residence area**	North	36.6 (35.7-37.4)	43.9 (43.3-44.7)	19.5 (18.8-20.1)	0.004
		Centre	36.0 (34.7-37.4)	44.3 (43.1-45.4)	19.7 (18.8-20.7)	
		South	38.4 (37.3-39.5)	41.8 (40.8-42.9)	19.8 (18.9-20.7)	
	**Parents' educational level****	High level of education	38.5 (37.4-39.6)	42.5 (41.5-43.6)	19.0 (18.2-19.8)	0.000
		Medium level of education	38.1 (37.2-39.0)	43.7 (42.8-44.6)	18.2 (17.5-18.9)	
		Low level of education	37.6 (35.8-39.5)	43.1 (41.2-44.9)	19.3 (17.8-20.8)	
		Don't know	34.0 (32.9-35.1)	43.3 (42.1-44.4)	22.7 (21.8-23.7)	
	**Parents' nationality**	One foreign parent	36.7 (34.9-38.5)	43.7 (41.9-45.6)	19.6 (18.2-21.0)	0.002
		Both Italian parents	37.5 (36.8-38.2)	43.1 (42.5-43.8)	19.4 (18.9-20.0)	
		Both foreign parents	35.7 (34.2-37.1)	42.8 (41.4-44.3)	21.5 (20.3-22.7)	
	**Family Affluence Scale (FAS)**	High	34.8 (33.6-36.0)	42.9 (41.6-44.1)	22.3 (21.3-23.4)	0.000
		Medium	37.3 (36.5-38.1)	43.7 (42.9-44.4)	19.0 (18.4-19.7)	
		Low	38.6 (37.6-39.7)	43.1 (41.9-44.1)	18.3 (17.5-19.2)	

** The highest educational level between the two parents.

Table S2. Logistic to predict *negative* influence of COVID-19 pandemic.

	Dimensions of adolescents' lives									
	Life in general	***Overall health***	***Relationship with family***	***Relationship with friends***	***Mental health***	***School performance***	***Phisical activity***	***Eating behaviour***	***Future expectation***	***Family finances***
	n=83,450	n=83,493	n=83,384	n=83,391	n=83,548	n=83,543	n=83,231	n=83,135	n=83,260	n=83,170
	OR (95% CI)	OR (95% CI)	OR (95% CI)	OR (95% CI)	OR (95% CI)	OR (95% CI)	OR (95% CI)	OR (95% CI)	OR (95% CI)	OR (95% CI)
**Age**										
11-years old (ref)										
13-years old	0.97 (0.90-1.05)	1.09 (0.99-1.20)	**1.34 (1.21-1.48)**	0.96 (0.89-1.05)	1.49 (1.39-1.61)***	**1.40 (1.28-1.53)**	**1.19 (1.11-1.28)**	**1.32 (1.21-1.44)**	**1.35 (1.24-1.48)**	**1.16 (1.06-1.28)**
15-years old	**1.29 (1.20-1.40)**	**1.46 (1.33-1.60)**	**1.44 (1.29-1.59)**	**1.17 (1.08-1.28)**	**2.25 (2.08-2.43)**	**2.02 (1.85-2.20)**	**1.38 (1.28-1.48)**	**1.64 (1.51-1.79)**	**1.9 (1.75-2.07)**	**1.54 (1.41-1.69)**
17-years old	**1.55 (1.43-1.68)**	**1.77 (1.61-1.94)**	**1.32 (1.19-1.47)**	0.94 (0.86-1.03)	**3.00 (2.76-3.25)**	**1.92 (1.76-2.10)**	**1.20 (1.12-1.29)**	**1.65 (1.52-1.80)**	**2.32 (2.12-2.53)**	**1.73 (1.57-1.90)**
**Adolescent's sex**										
Male (ref)										
Female	**1.32 (1.26-1.39)**	**1.31 (1.24-1.39)**	**1.62 (1.51-1.74)**	**1.21 (1.15-1.28)**	**2.63 (2.50-2.77)**	0.95 (0.90-1.01)	**1.07 (1.02-1.12)**	**1.74 (1.64-1.84)**	**1.52 (1.44-1.60)**	1.01 (0.95-1.07)
**Residence area**										
North (ref)										
Centre	0.94 (0.88-1.00)	0.95 (0.88-1.02)	0.95 (0.88-1.03)	**0.85 (0.79-0.91)**	**0.93 (0.87-0.99)**	**0.89 (0.83-0.96)**	0.97 (0.92-1.03)	0.99 (0.92-1.05)	**0.85 (0.79-0.90)**	1.02 (0.95-1.07)
South	1.06 (1.00-1.12)	0.99 (0.92-1.06)	**0.92 (0.85-0.99)**	**0.79 (0.74-0.84)**	0.95 (0.89-1.01)	**0.93 (0.87-0.99)**	1.00 (0.95-1.06)	1.02 (0.96-1.09)	**0.82 (0.77-0.88)**	**0.87 (0.81-0.93)**
**Parent's educational level**										
Low level (ref)										
Medium level	1.06 (0.96-1.15)	1.10 (0.99-1.22)	1.06 (0.93-1.20)	1.06 (0.96-1.17)	**1.23 (1.12-1.34)**	**0.86 (0.78-0.95)**	1.07 (0.98-1.17)	1.07 (0.97-1.19)	1.04 (0.94-1.15)	0.97 (0.87-1.08)
High level	**1.16 (1.06-1.28)**	**1.14 (1.02-1.28)**	**1.25 (1.09-1.44)**	**1.28 (1.14-1.42)**	**1.38 (1.25-1.52)**	**0.84 (0.76-0.93)**	**1.19 (1.08-1.31)**	**1.17 (1.05-1.30)**	**1.11 (1.00-1.24)**	**0.76 (0.68-0.86)**
Don't know	1.01 (0.92-1.11)	**1.36 (1.21-1.53)**	**1.30 (1.14-1.49)**	**1.13 (1.02-1.26)**	**1.14 (1.03-1.26)**	1.02 (0.92-1.14)	**1.13 (1.02-1.24)**	1.06 (0.95-1.19)	**1.25 (1.12-1.39)**	0.96 (0.85-1.08)
**Parent's nationality**										
Both italian (ref)										
One foreign	0.98 (0.90-1.06)	0.98 (0.89-1.09)	**1.13 (1.01-1.28)**	1.00 (0.91-1.10)	1.00 (0.92-1.08)	**1.26 (1.15-1.38)**	0.96 (0.89-1.04)	0.94 (0.86-1.03)	**1.10 (1.00-1.21)**	**1.20 (1.08-1.34)**
Both foreign	0.98 (0.91-1.05)	**1.15 (1.05-1.26)**	**1.27 (1.16-1.40)**	1.00 (0.92-1.08)	**0.89 (0.82-0.96)**	**1.37 (1.27-1.49)**	0.96 (0.89-1.04)	1.07 (0.98-1.16)	**1.13 (1.04-1.22)**	**1.35 (1.23-1.48)**
**Family Affluence Scale (FAS)**										
Low (ref)										
Medium	**0.94 (0.89-0.99)**	0.97 (0.91-1.04)	**0.83 (0.77-0.90)**	1.01 (0.95-1.07)	0.97 (0.92-1.03)	**0.85 (0.79-0.90)**	0.97 (0.91-1.02)	0.95 (0.89-1.02)	**0.89 (0.84-0.95)**	**0.62 (0.58-0.67)**
High	**0.85 (0.39-0.48)**	**0.89 (0.82-0.98)**	**0.84 (0.76-0.92)**	**0.88 (0.81-0.96)**	**0.84 (0.78-0.91)**	**0.81 (0.76-0.89)**	**0.80 (0.75-0.86)**	**0.91 (0.84-0.99)**	**0.80 80.74-0.87)**	**0.47 (0.43-0.53)**

***statistically significant results are in bold**

Table S3. Logistic to predict *positive* influence of COVID-19 pandemic.

	Dimensions of adolescents' lives									
	Life in general	***Overall health***	***Relationship with family***	***Relationship with friends***	***Mental health***	***School performance***	***Phisical activity***	***Eating behaviour***	***Future expectation***	***Family finances***
	n=83,450	n=83,493	n=83,384	n=83,391	n=83,548	n=83,543	n=83,231	n=83,135	n=83,260	n=83,170
	OR (95% CI)	OR (95% CI)	OR (95% CI)	OR (95% CI)	OR (95% CI)	OR (95% CI)	OR (95% CI)	OR (95% CI)	OR (95% CI)	OR (95% CI)
**Age**										
11-years old (ref)										
13-years old	**0.78 (0.72-0.84)**	**0.61 (0.57-0.66)**	**0.58 (0.57-0.62)**	**0.77 (0.72-0.83)**	**0.62 (0.57-0.67)**	**0.72 (0.67-0.78)**	**0.69 (0.65-0.75)**	**0.69 (0.64-0.74)**	**0.64 (0.59-0.69)**	**0.60 (0.56-0.64)**
15-years old	**0.56 (0.52-0.61)**	**0.37 (0.34-0.40)**	**0.39 (0.36-0.42)**	**0.57 (0.52-0.61)**	**0.38 (0.34-0.41)**	**0.54 (0.50-0.58)**	**0.57 (0.53-0.62)**	**0.51 (0.48-0.55)**	**0.39 (0.36-0.43)**	**0.36 (0.33-0.39)**
17-years old	**0.49 (0.45-0.54)**	**0.29 (0.26-0.31)**	**0.36 (0.33-0.39)**	**0.55 (0.51-0.60)**	**0.30 (0.27-0.33)**	**0.56 (0.51-0.60)**	**0.63 (0.59-0.69)**	**0.44 (0.40-0.47)**	**0.33 (0.31-0.36)**	**0.26 (0.24-0.28)**
**Adolescent's sex**										
Male (ref)										
Female	**0.63 (0.59-0.67)**	**0.80 (0.76-0.84)**	**0.78 (0.74-0.82)**	**0.81 (0.77-0.85)**	**0.46 (0.44-0.49)**	**0.93 (0.88-0.97)**	**0.76 (0.72-0.80)**	**0.70 (0.67-0.74)**	**0.76 (0.72-0.80)**	**0.89 (0.85-0.94)**
**Residence area**										
North (ref)										
Centre	1.04 (0.97-1.12)	**1.11 (1.05-1.19)**	**1.13 (1.07-1.20)**	**1.22 (1.15-1.30)**	**1.07 (1.00-1.15)**	1.02 (0.99-1.12)	1.05 (0.99-1.12)	**1.13 (1.06-1.20)**	**1.20 (1.13-1.28)**	**1.12 (1.05-1.19)**
South	1.05 (0.98-1.13)	**1.25 (1.17-1.33)**	**1.34 (1.26-1.42)**	**1.43 (1.35-1.51)**	**1.15 (1.08-1.23)**	**1.14 (1.08-1.21)**	**1.09 (1.02-1.15)**	**1.33 (1.25-1.41)**	**1.53 (1.44-1.63)**	**1.47 (1.38-1.56)**
**Parent's educational level**										
Low level (ref)										
Medium level	0.92 (0.83-1.03)	**0.83 (0.76-0.91)**	**0.91 (0.83-0.99)**	0.96 (0.88-1.05)	**0.88 (0.79-0.97)**	**1.18 (1.09-1.29)**	0.91 (0.83-1.00)	**0.78 (0.71-0.86)**	0.95 (0.86-1.04)	0.91 (0.83-1.00)
High level	**0.86 (0.77-0.97)**	**0.74 (0.67-0.82)**	**0.77 (0.70-0.84)**	**0.79 (0.72-0.87)**	**0.81 (0.73-0.90)**	**1.20 (1.09-1.31)**	**0.86 (0.78-0.95)**	**0.67 (0.61-0.74)**	**0.81 (0.73-0.90)**	**0.89 (0.81-0.99)**
Don't know	0.95 (0.84-1.07)	**0.88 (0.80-0.97)**	**0.76 (0.69-0.85)**	**0.87 (0.80-0.96)**	0.94 (0.85-1.05)	0.94 (0.87-1.02)	**0.88 (0.79-0.97)**	**0.83 (0.74-0.92)**	**0.80 (0.71-0.89)**	**0.88 (0.79-0.98)**
**Parent's nationality**										
Both italian (ref)										
One foreign	1.00 (0.90-1.11)	**1.06 (0.97-1.15)**	**0.91 (0.84-0.99)**	1.05 (0.96-1.14)	1.08 (0.98-1.19)	0.94 (0.87-1.02)	1.03 (0.94-1.12)	1.02 (0.93-1.11)	0.97 (0.89-1.07)	0.99 (0.90-1.08)
Both foreign	**1.11 (1.02-1.22)**	**1.13 (1.05-1.22)**	0.98 (0.91-1.05)	1.02 (0.95-1.10)	**1.27 (1.17-1.38)**	**0.77 (0.71-0.83)**	1.0 (0.92-1.09)	**1.21 (1.12-1.31)**	**1.11 (1.03-1.20)**	**1.15 (1.06-1.24)**
**Family Affluence Scale (FAS)**										
Low (ref)										
Medium	1.06 (0.99-1.14)	0.97 (0.92-1.03)	**1.06 (1.00-1.12)**	1.01 (0.95-1.07)	1.05 (0.99-1.12)	**1.15 (1.09-1.22)**	**1.19 (1.12-1.26)**	0.97 (0.91-1.03)	**1.08 (1.01-1.14)**	**1.32 (1.24-1.40)**
High	**1.27 (1.17-1.39)**	**1.17 (1.09-1.26)**	**1.18 (1.10-1.26)**	**1.18 (1.09-1.27)**	**1.25 (1.15-1.35)**	**1.24 (1.15-1.33)**	**1.53 (1.42-1.65)**	**1.14 (1.06-1.23)**	**1.30 (1.22-1.42)**	**2.06 (1.91-2.22)**

***statistically significant results are in bold**
